# Heat-Killed *Enterococcus faecalis* EF-2001 Ameliorates Atopic Dermatitis in a Murine Model

**DOI:** 10.3390/nu8030146

**Published:** 2016-03-05

**Authors:** Eun-Ju Choi, Masahiro Iwasa, Kwon-Il Han, Wan-Jae Kim, Yujiao Tang, Young Joung Hwang, Jeong Ryong Chae, Weon Cheol Han, Yu-Su Shin, Eun-Kyung Kim

**Affiliations:** 1 Division of Sport Science, College of Science and Technology, Konkuk University, Chungju 380-701, Korea; ooj7990@kku.ac.kr; 2 Research & Development Center, Korea BeRM Co. Ltd., Seoul 135-878, Korea; masa@brm.co.jp (M.I.); kihan@berm.co.kr (K.-I.H.); wanjae74@berm.co.kr (W.-J.K.); 3 Division of Food BioScience, College of Biomedical and Health Sciences, Konkuk University, Chungju 380-701, Korea; yuanxi00@kku.ac.kr; 4 Department of Food Science & Culinary, International University of Korea, Jinju 660-759, Korea; hyjnara@kebi.com; 5 Department of Physical Education, Department of Physical Education, Kunsan National University, Kunsan 54150, Korea; cjr@kunsan.ac.kr; 6 Department Pathology, Sanbon Medical Center, Wonkwang University, Sanbon 435-040, Korea; bladeroyal@naver.com; 7 Department of Ginseng and Medicinal Herb National Institute of Horticulture Herbal Science, RDA, Chungbuk 27709, Korea; totoro69@korea.kr

**Keywords:** atopic dermatitis, lactic acid bacteria, heat-killed *Enterococcus faecalis* EF-2001, immunomodulatory, pathogenic cytokines

## Abstract

Recent reports have shown the immunomodulatory effect of heat-killed lactic acid bacteria. Atopic dermatitis (AD) is an allergic skin disease, caused by immune dysregulation among other factors. The aim of this study was to assess the effect of heat-killed *Enterococcus faecalis* EF-2001 (EF-2001) on AD. We established an *in vivo* AD model by repeated local exposure of *Dermatophagoides farinae* extract (DFE; house dust mite extract) and 2,4-dinitrochlorobenzene (DNCB) to the ears of mice. After oral administration of EF-2001 for four weeks, the epidermal and dermal ear thickness, mast cell infiltration, and serum immunoglobulin levels were measured. In addition, the gene expression levels of pathogenic cytokines in the ears, lymph nodes, and splenocytes were assayed. EF-2001 attenuated AD symptoms based on the ear thickness, histopathological analysis, and serum immunoglobulin levels. Moreover, EF-2001 decreased the DFE/DNCB-induced expression of various pathogenic cytokines in the ears, lymph nodes, and splenocytes. These results suggest that EF-2001 has therapeutic potential in the treatment of AD owing to its immunomodulatory effects.

## 1. Introduction

Many probiotic bacteria have been described as promising agents for the treatment and prevention of atopic dermatitis (AD) by influencing the host’s immune system [1,2]. Most of these bacteria are lactic acid bacteria, which form part of the healthy human microbiota. However, emerging safety concerns about the extensive use of live microbial cells, such as shelf-life problems and the risks of microbial translocation and infection, have led to enhanced research interest in the use of non-viable microorganisms or microbial cell extracts [3].

Recent research findings have provided insight into the complex pathogenic mechanisms involved in AD [4]. The pathogenesis of AD involves immune dysregulation [5], marked epidermal hyperplasia in chronic lesional skin [6], and increased susceptibility to infections [7]. Although the exact pathogenesis of AD is not yet completely understood, it is generally characterized by excessive T-cell activation, with significant skin infiltration by T-cells [4]. Activation of both T helper type (Th)2 (interleukin (IL)-4 and IL-5) and Th22 (IL-22) cells is a hallmark of AD, with some contribution of Th17 (IL-17) and Th1 (interferon (INF)-γ) components [8]. An important breakthrough in the understanding of the pathogenesis of AD was provided by studies investigating the genomic and histological profiles of AD skin [9,10]. These include markers of epidermal hyperplasia, as well as markers of cellular infiltrates.

*Enterococcus faecalis*, a gram-positive commensal microorganism in the intestines was investigated to have immuno-stimulatory or-regulatory activities (*Enterococcus faecalis* FK-23) [11,12]. However the microorganism undergoes a change from commensal to pathogenic with its shift from the gastrointestinal tract to the bloodstream potentially causing life-threatening infections, such as bacterial endocarditis or another systemic infection related to septicemia [13,14].

Meanwhile, heat-killed *Enterococcus faecalis* derived from the intestines was reported to have a radiation protection effect and antitumor activity despite having been heat killed [15,16]. However, to the best of our knowledge, little is known about the role of heat-killed *Enterococcus faecalis* EF-2001 on AD in a murine model. The aim of this study was to investigate the immunomodulatory properties of non-viable probiotic *Enterococcus faecalis* EF-2001 on AD, in order to clarify the complex pathogenic mechanism of this disease *in vivo*, using a murine model.

## 2. Materials and Methods

### 2.1. Animals

Eight-week-old female BALB/c mice were purchased from Samtako and housed under specific pathogen-free conditions. All experiments were approved by the Institutional Animal Care and Use Committee of Konkuk University (KU13074).

### 2.2. EF-2001

EF-2001 is a commercially available probiotic that was originally isolated from healthy human feces. It was supplied as a heat-killed and dried powder from Nihon BRM Co. Ltd (Tokyo, Japan). One gram of dried EF-2001 is equivalent to 7.5 × 10^12^ colony-forming units prior to being heat-killed.

### 2.3. Induction of AD Lesions in the Ear

**Figure 1:**
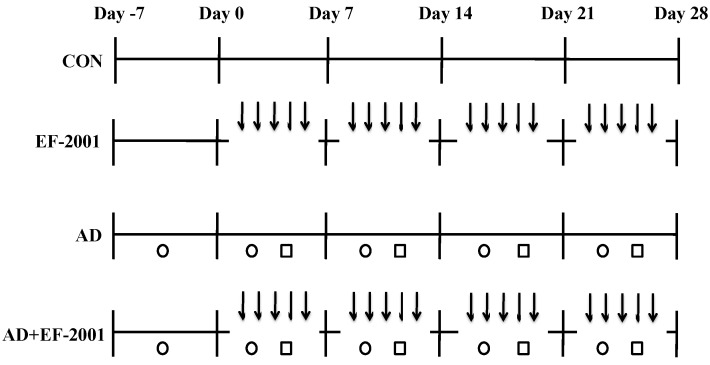
Experimental schedule for the induction of atopic dermatitis (AD) lesions. After seven days of initial boosting with 2,4-dinitrochlorobenzene (DNCB), DNCB and *Dermatophagoides farinae* extract (DFE) were applied to both ears once a week for four weeks. Starting at one week following the first induction, heat-killed *Enterococcus faecalis* EF-2001 (EF-2001; 2 mg/kg) was orally administered daily for four weeks. ↓, EF-2001; ○, DNCB; □, DFE; CON, control.

AD was induced in mice by repeated local exposure of *Dermatophagoides farinae* extract (DFE; house dust mite extract) and 2,4-dinitrochlorobenzene (DNCB) to the ears, as previously described [17]. A schematic of the experimental procedure is provided in Figure 1. For the induction of AD, the mice were divided into four groups (control, AD-only, EF-2001-only, and AD + EF-2001), and the surfaces of both earlobes were stripped five times with surgical tape (Nichiban, Tokyo, Japan). After stripping, 20 µL of 1% DNCB was painted onto each ear, followed by 20 µL of DFE (10 mg/mL) four days later. DFE and DNCB treatment was administered once a week for four weeks. Animals received EF-2001 (2 mg/kg orally administered) throughout the four weeks of AD induction.

The ear thickness was measured 24 h after DNCB or DFE application with a dial thickness gauge (Kori Seiki MFG, Co., Tokyo, Japan). At days 14 and 28, blood samples were collected by orbital puncture. Plasma samples were prepared from the cardiac puncture under ketamine/xylazine anesthesia and stored at −70 °C for further analysis. After blood collection, the ears were removed and used for histopathological analysis. The serum immunoglobulin (Ig)E, DFE specific IgE and IgG2a levels were measured at days 14 and 28 after the first induction using an IgE enzyme-linked immunoassay kit (Bethyl Laboratories, Inc., Montgomery, TX, USA) according to the manufacturer’s instructions.

### 2.4. Histological Observations

Excised ears were fixed in 4% paraformaldehyde for 16 h and embedded in paraffin. Thin (6 µm) sections were stained with hematoxylin and eosin (H & E). The thickness of the epidermis and dermis was measured under a microscope. For measurement of mast cell infiltration, skin sections were stained with toluidine blue and the number of mast cells was counted in five randomly chosen fields of view.

### 2.5. Preparation of Splenocytes

Splenocytes were harvested as described in Gao *et al.* [18]. The spleen was removed at the time of killing and a single-cell suspension was prepared by forcing the spleen through a 400 μm stainless steel mesh. Erythrocytes were lysed with hypotonic buffered solution and lymphocytes were washed with Hanks’ balanced salt solution (HBSS) prior to being resuspended in RPMI 1640 medium. Viable cells were counted by the trypan blue exclusion method using a hemocytometer. Finally, cells were adjusted into different concentrations with RPMI 1640 medium supplemented with 10% fetal calf serum.

### 2.6. Real-Time Polymerase Chain Reaction (PCR)

The total RNA was isolated from cells using TRIzol according to the manufacturer’s protocol. The first-strand complementary DNA (cDNA) was synthesized using Superscript II reverse transcriptase (Invitrogen, Waltham, MA, USA). Quantitative real-time PCR was carried out using a Thermal Cycler Dice TP850 (Takarabio Inc., Shiga, Japan) according to the manufacturer’s protocol. Total RNA was isolated from the ear tissues, cervical lymph nodes, and splenocytes of each group. The conditions for PCR were similar to those previously described [17]. Briefly, 2 μL of cDNA (100 ng), 1 µL of sense and antisense primer solution (0.4 µM), 12.5 µL of SYBR Premix Ex Taq (Takarabio Inc., Kusatsu, Shiga, Japan), and 9.5 µL of dH_2_O were mixed to obtain a final 25 µL reaction mixture in each reaction tube. The primers used for PCR were: mouse tumor necrosis factor-α (*TNF-α*), 5′-AAGCCTGTAGCCCACGTCGTA-3′ and 5′-GGCACCACTAGTTGGTTGTCTTTG-3′; mouse *IFN-γ*, 5′-TCAAGTGGCATAGATGTGGAAGAA-3′ and 5′-TGGCTCTGCAGGATTTTCATG-3′; mouse *IL-4*, 5′-ACAGGAGAAGGGACGCCAT-3′ and 5′-GAAGCCGTACAGACGAGCTCA-3′; mouse *IL-5*, 5′-GAAGTGTGGCGAGGAGAGAC-3′ and 5′-GCACAGTTTTGTGGGGTTTT-3′; mouse *IL-17*, 5′-CCTACCAGACCAAGGTCAAC-3′ and 5′-AGGGGGTAATAAAGGGATTG-3′; mouse *IL-22*, 5′-TCCCTCTGTCATCTGGGAAG-3′ and 5′-CTCGACCCTGAAAGTGAAGG-3′; mouse *IL-31*, 5′-TCGGTCATCATAGCACATCTGGAG-3′ and 5′-GCACAGTCCCTTTGGAGTTAAGTC-3′; mouse *IL-32*, 5′-AGCAAGGACGGCGAATGTT-3′ and 5′-GGGTGGACATATAAGCGGTTC-3′; mouse thymic stromal lymphopoietin (*TSLP)*, 5′-CTCGGCCATTCGTACATGGAA-3′ and 5′-GGATACCTC TGCACCGTAGC-3′; mouse glyceraldehyde 3-phosphate dehydrogenase (*GAPDH)*, 5′-GCACAGTCAAGGCCGAGAAT-3′ and 5′-GCCTTCTCCATGGTGGTGAA-3′. The amplification conditions were 10 s at 95 °C, 40 cycles of 5 s at 95 °C and 30 s at 60 °C, 15 s at 95 °C, 30 s at 60 °C, and 15 s at 95 °C. The mRNA levels of the target genes, relative to *GAPDH*, were normalized using the following formula: relative mRNA expression = 2^−(ΔCt of target gene − ΔCt of *GAPDH*)^, where Ct is the threshold cycle value. In each sample, the expression of the analyzed gene was normalized to that of *GAPDH* and presented as the relative mRNA level.

### 2.7. Statistical Analysis

Statistical analysis of data was carried out using the SAS statistical software (SAS Institute, Cary, NC, USA). Multiple group data were analyzed using one-way analysis of variance followed by Dunnett’s multiple range tests. All results are expressed as the mean ± standard deviation of comparative fold differences. Data are representative of three independent experiments. Significance was set at *p* < 0.05.

## 3. Results

### 3.1. Effect of EF-2001 on the Ear Thickness and Histopathological Observation

**Figure 2:**
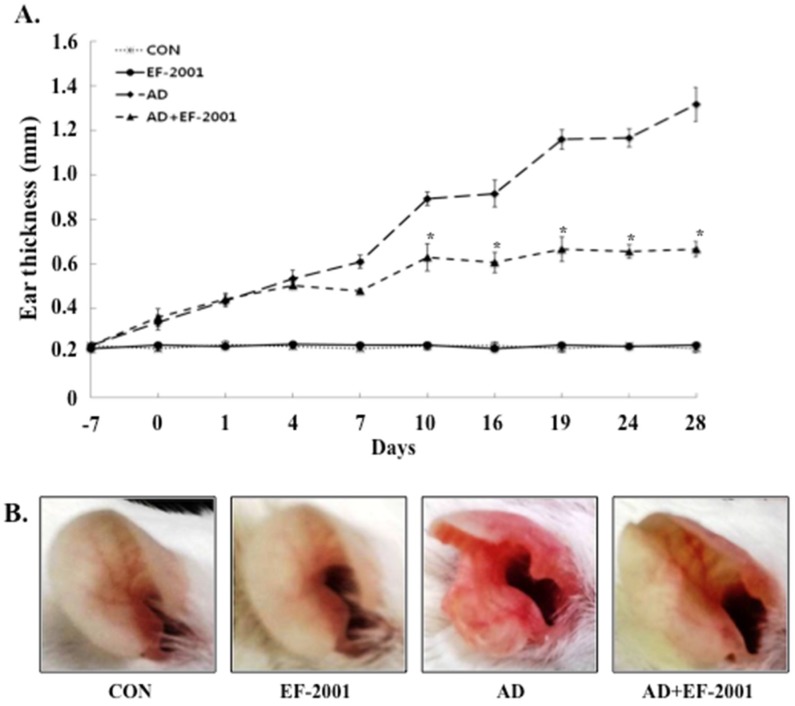

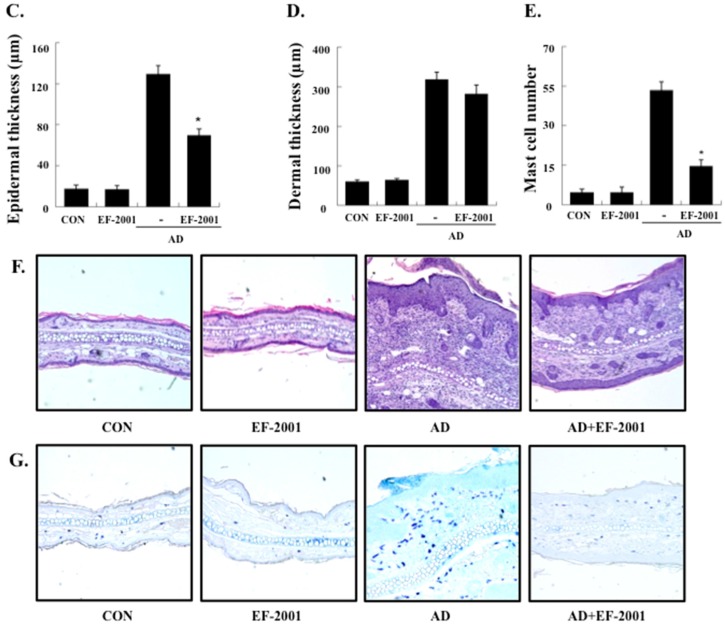
Histopathological analysis to assess the effect of EF-2001 on the ear thickness and mast cell infiltration. (**A**) The ear thickness was measured 24 h after 2,4-dinitrochlorobenzene (DNCB) or *Dermatophagoides farinae* extract (DFE) application with a dial thickness gauge; (**B**) Photographs of the ears of mice from each group on day 28; Epidermal (**C**) and dermal (**D**) thickness was measured from hematoxylin and eosin-stained microphotographs; (**E**) The number of infiltrated mast cells was determined on the basis of toluidine blue staining; Representative photomicrographs of ear sections stained with hematoxylin and eosin (**F**) or toluidine blue (**G**). Data are presented as the mean ± SD of six determinations of each group (*n* = 6). * Significant difference from the DFE/DNCB-treated value at *p* < 0.05. The pictures shown are representative of each group (*n* = 3–6). Original magnification was 200×. CON, control; EF-2001, heat-killed *Enterococcus faecalis* EF-2001; AD, atopic dermatitis

To investigate the effect of EF-2001 on AD, a BALB/c AD model was established by painting DFE and DNCB on both earlobes for four weeks [17]. As shown in Figure 2A,B, repeated topical application of DFE/DNCB significantly increased the ear thickness in mice. In addition, EF-2001 reduced the DPE/DNCB-induced increase in ear thickness. DFE/DNCB also induced remarkable AD lesions, such as hemorrhage, edema, excoriation, and scaling, which were diminished by EF-2001 treatment (Figure 2B).

To analyze the effect of EF-2001 on skin hypertrophy and granulocyte infiltration, sections of the ear were stained and observed under an optical microscope. Repeated DFE/DNCB exposure caused potent inflammatory changes such as thickening of the epidermis and dermis, fibrosis in the dermis, and accumulation of inflammatory cells such as lymphocytes, eosinophils, and neutrophils in the ear tissue of the AD mice (Figure 2C,D,F).

To further investigate these changes, we evaluated the effects of EF-2001 on the infiltration of mast cells, an important effector cell and the primary source of histamine involved in AD, into the ears. Indeed, EF-2001 treatment reduced the infiltration of mast cells induced by DFE/DNCB (Figure 2E,G).

### 3.2. Effect of EF-2001 on Serum Ig Levels

**Figure 3:**
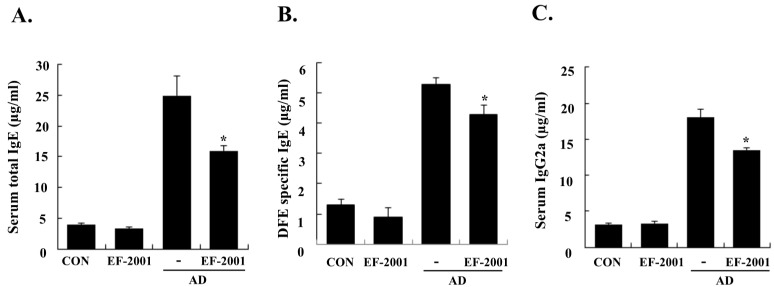
Effect of heat-killed *Enterococcus faecalis* EF-2001 (EF-2001) on serum immunoglobulin levels. The blood samples for the control (CON), EF-2001, *Dermatophagoides farinae* extract/2,4-dinitrochlorobenzene (DFE/DNCB), and EF-2001 plus DFE/DNCB groups were collected by orbital puncture at day 28, and plasma levels of total IgE (**A**); DFE specific IgE (**B**); and IgG2a (**C**) were quantified by enzyme-linked immunosorbent assay. Data are presented as mean ± SD of triplicate determinations of each group (*n* = 6). * Significant difference from the DFE/DNCB-treated value at *p* < 0.05. AD: atopic dermatitis induced by DFE and DNCB treatment.

To determine whether EF-2001 exerts its effects mainly via the Th1 or Th2 response, the serum levels of IgE (total and DFE-specific) and IgG2a were examined. Repeated application of DFE/DNCB caused an apparent elevation of total IgE, DFE-specific IgE, and IgG2a. However, EF-2001 significantly reduced the DFE/DNCB-induced serum levels of total IgE, DFE-specific IgE, and IgG2a (Figure 3).

### 3.3. Effect of EF-2001 on the Expression of Various Pathogenic Cytokines

**Figure 4:**
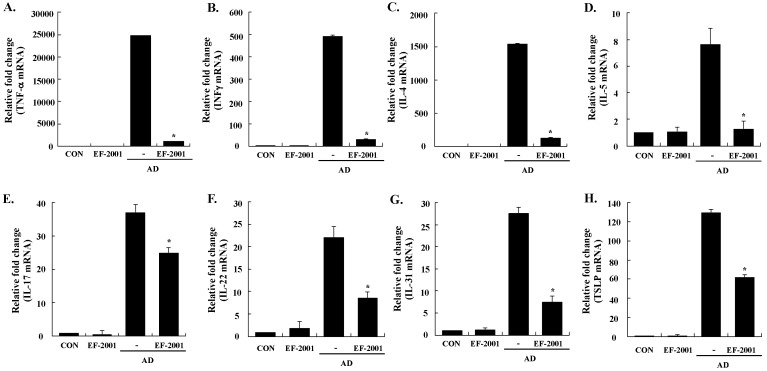
Effect of heat-killed *Enterococcus faecalis* EF-2001 (EF-2001) on the expression of various pathogenic factors in the ear. **A**, TNF-α; **B**, INF-γ; **C**, IL-4; **D**, IL-5; **E**, IL-17; **F**, IL-22; **G**, IL-31; **H**, TSLP. The ears were excised on day 28 and total RNA was isolated. Quantitative real-time polymerase chain reaction was performed as described in the Methods. Data are presented as mean ± SD of triplicate determinations of each group (*n* = 6). * Significant differences from the DFE/DNCB-treated value at *p* < 0.05. DFE, *Dermatophagoides farinae* extract; DNCB, 2,4-dinitrochlorobenzene; AD: atopic dermatitis induced by DFE and DNCB treatment.

**Figure 5:**
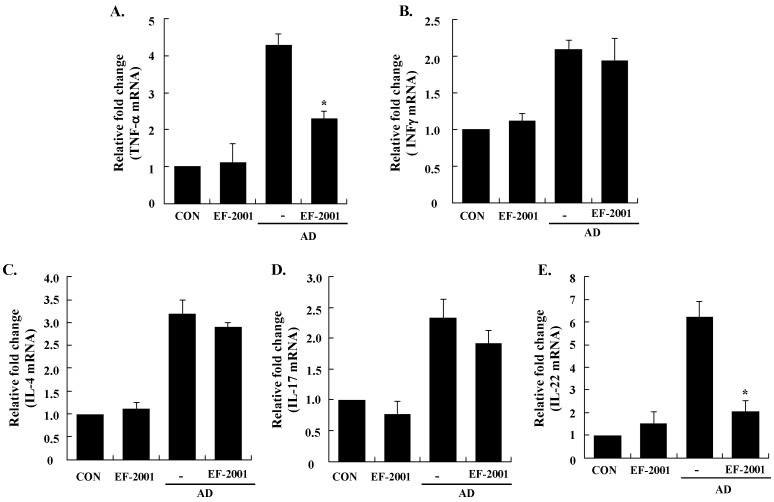
Effect of heat-killed *Enterococcus faecalis* EF-2001 (EF-2001) on the expression of various pathogenic factors in the cervical lymph nodes. **A**, TNF-α; **B**, INF-γ; **C**, IL-4; **D**, IL-17; **E**, IL-22. The ears were excised on day 28 and total RNA was isolated. Quantitative real-time polymerase chain reaction was performed as described in the Methods. Data are presented as the mean ± SD of triplicate determinations of each group (*n* = 6). * Significant differences from the DFE/DNCB-treated value at *p* < 0.05. DFE, *Dermatophagoides farinae* extract; DNCB, 2,4-dinitrochlorobenzene; AD: atopic dermatitis induced by DFE and DNCB treatment.

**Figure 6:**
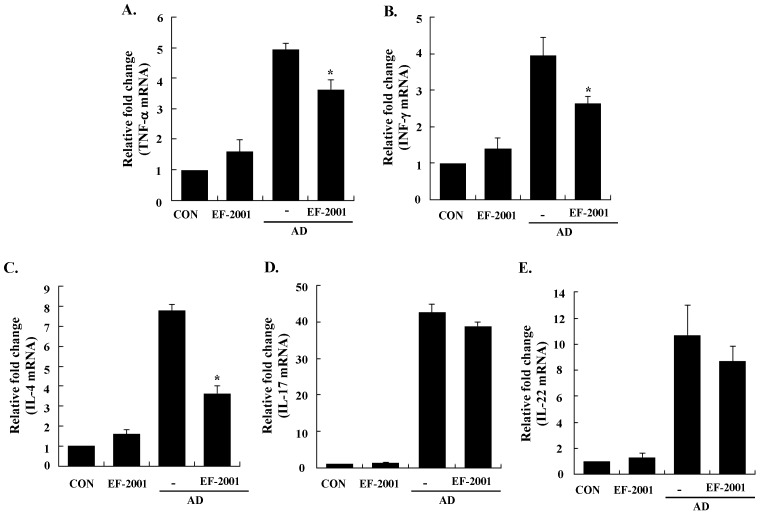
Effect of heat-killed *Enterococcus faecalis* EF-2001 (EF-2001) on the expression of various pathogenic factors in the splenocytes. **A**, TNF-α; **B**, INF-γ; **C**, IL-4; **D**, IL-17; **E**, IL-22. The ears were excised on day 28 and total RNA was isolated. Quantitative real-time polymerase chain reaction was performed as described in the Methods. Data are presented as the mean ± SD of triplicate determinations. * Significant differences from the DFE/DNCB-treated value at *p* < 0.05. DFE, *Dermatophagoides farinae* extract; DNCB, 2,4-dinitrochlorobenzene; AD: atopic dermatitis induced by DFE and DNCB treatment.

Next, to understand the mechanism of EF-2001 in alleviating the AD response, we examined the expression levels of AD-related inflammatory cytokines from the ear tissue, cervical lymph nodes, and splenocytes by real-time PCR. All of the cytokines tested were upregulated in the ear tissue, cervical lymph nodes, and splenocytes of AD mice, and EF-2001 inhibited the expression of pro-inflammatory (*TNF-α*), Th1 (*IFN-γ*), Th2 (IL-4, IL-5, and IL-31), IL-17, and IL-22 cytokines, and *TSLP* from the ear, cervical lymph node, or splenocyte tissues (Figure 4, Figure 5 and Figure 6). In addition, EF-2001 itself did not affect the cytokines expressions. These results suggest that EF-2001 inhibits the expression of both Th1 and Th2 cytokines in AD from the ear, cervical lymph node, and splenocyte tissue.

## 4. Discussion

Probiotics are microorganisms that provide health benefits when consumed. It has been suggested that administration of probiotics may have preventive and/or therapeutic potential in AD [1,2]. Although the therapeutic benefits of probiotics have been observed for more than a century, safety issues have not yet been definitively clarified. There are case reports of complications resulting from specific bacteria therapy; this suggests that revising probiotic safety aspects may be warranted [19,20,21]. In individuals with a pre-existing structural heart disease or severe immunodeficiency, lactobacilli can translocate and cause severe infection [22]. By contrast, non-viable microorganisms or microbial cell extracts could eliminate the shelf-life problems and reduce the risks of microbial translocation and infection associated with live probiotics. In particular, non-viable or heat-killed lactic acid bacteria not only provide the advantages of longer product shelf life, more convenient transportation and easier storage, but also immunomodulatory functions [23]. Recently, non-viable lactobacilli (*Lactobacillus plantarum* L-137, *Lactobacillus rhamnosus* GG, *Lactobacillus acidophilus* L-92) were reported to show similar beneficial immunomodulatory effects in experimental animals [24,25] and humans [26]. However, the specific health benefits of non-viable probiotics administered during AD are less well established. In this study, to elucidate the effects of non-viable probiotics in the modulation of immunological systems, we studied the effect of EF-2001 on AD lesions using a BALB/c model. The effects of EF-2001 on alleviating AD symptoms and stimulating the immune system were evaluated by measuring the ear thickness, histopathological changes (including mast cell infiltration), serum Ig levels, and gene expression levels of AD-related pathogenic cytokines in the ear tissue, cervical lymph nodes, and splenocytes in the AD model mice.

Mast cells release several important signaling molecules, among which histamine has particularly potent pro-inflammatory activities [27]. In the present study, EF-2001 mitigated the typical and histological changes of AD, such as severe ear thickness, ulcers, epidermal thickness, epidermal hyperplasia, and infiltration of mast cells (Figure 2). Oral administration of EF-2001 significantly reduced the typical histopathological phenomena of AD, and the number of mast cells infiltrating the skin lesions of the AD mice.

In AD patients, elevated total IgE and IgE specific to environmental allergens is commonly detected [28]. Hyper-production of IgE is associated with the Th2 cellular response, and is a major characteristic of allergic disorders including AD. By contrast, IgG2a production is associated with the Th1 response [29]. Historically, AD was considered to result from a Th1/Th2 imbalance. Th1-mediated inflammation serves to fight intracellular infections through its main cytokine, IFN γ, whereas Th2-associated cytokines such as IL-4 and IL-5 are involved to fight extracellular pathogens and in allergic responses and mediate IgE class switching, among other functions [30,31]. Levels of IL-31 also correlate with levels of Th2 cells in the skin of subjects with AD [32]. In the present study, EF-2001 decreased IL-5 and IL-31 levels of the ear tissue, which play an important role in Ig isotype switching. These results imply that EF-2001 could suppress the elevation of serum Ig levels by decreasing the Th2 response, especially IL-4 and IL-5 production in the ear tissue (Figure 3 and Figure 4C,D). Recently, Th17 and Th22 were identified as distinct T-cell subsets involved in the pathogenesis of various conditions, including allergic skin diseases [33,34,35,36,37]. In AD patients, the number of peripheral blood IL-17+CD4+ T cells correlates with disease severity [38]. In the skin, IL-22 induces keratinocyte proliferation and epidermal hyperplasia, and the frequency of IL-22-expressing T cells in AD skin is correlated with disease severity [39,40]. In addition, TSLP is a novel cytokine involved in the immunopathogenesis of AD [41]. In the ear tissue, TNF-α, IFN-γ, IL-4, IL-5, IL-17, IL-22, and IL-31 were markedly inhibited by EF-2001. However, in the cervical lymph nodes or splenocytes, only some of them were inhibited by EF-2001. We assume the reason was that since AD was directly induced on the ear tissue, more cytokines of the ear tissue were affected than ones of the cervical lymph node or splenocyte. Previous studies have reported that heat-killed *Lactobacillus casei* Shirota*,* suppressed pro-inflammatory, Th1, and Th2 cytokines in splenocytes [42]. Similarly, we suggest that EF-2001 can significantly inhibit the inflammatory response by blocking both Th1 and Th2 in the cervical lymph nodes and splenocytes, as well as in the AD lesions of the ear tissue.

In summary, we demonstrated that EF-2001 relieved the development of DFE/DNCB-induced AD signs in BALB/c mice, decreasing histopathological signs, production of Ig, and the expression of pathogenic cytokines. We provide evidence that EF-2001 could be a potential therapeutic candidate for AD. Furthermore, our data suggest that EF-2001 may effectively prevent AD, and can be a useful pharmacological agent or food supplement.
